# Renal resistive index in patients with polycystic ovary syndrome

**DOI:** 10.20945/2359-3997000000141

**Published:** 2019-05-25

**Authors:** Gülsen Tüfekçioğlu, Şakir Özgür Keşkek, Okan Dilek, Cengiz Yilmaz

**Affiliations:** 1 Numune Training and Research Hospital Department of Internal Medicine Adana Turkey Department of Internal Medicine, Numune Training and Research Hospital, Adana, Turkey; 2 Numune Training and Research Hospital Department of Radiology Adana Turkey Department of Radiology, Numune Training and Research Hospital, Adana, Turkey

**Keywords:** Polycystic ovary syndrome, renal resistive index, cardiovascular disease, systolic blood pressure, insulin resistance

## Abstract

**Objective:**

Polycystic ovary syndrome (PCOS) is a common endocrine disorder in women of reproductive age. The renal resistive index (RRI) is a measure of renal arterial resistance to blood flow. The aim of this study was to investigate the renal resistive index levels of patients with PCOS.

**Subjects and methods:**

A total of 216 women were included in this cross-sectional study. The study group consisted of 109 patients with PCOS, and the control group consisted of 107 healthy subjects. The RRI of all subjects was measured using renal Doppler ultrasonography.

**Results:**

The patients with PCOS had higher RRI levels in comparison to the healthy subjects (0.64 ± 0.06 vs. 0.57 ± 0.06, p < 0.001). The RRI levels of the patients with PCOS were correlated with systolic blood pressure (p = 0.004, r = 0.268) and with homeostasis model assessment of insulin resistance (HOMA-IR) (p = 0.02, r = 0.216).

**Conclusion:**

In this study, we observed higher RRI levels in patients with PCOS. High RRI levels may be an indicator of cardiovascular and/or cardiovascular-associated diseases in patients with PCOS.

## INTRODUCTION

Polycystic ovary syndrome (PCOS) is a common endocrine disorder in women of reproductive age, and it is characterized by menstrual irregularities, signs of androgen excess, obesity, and hirsutism, as well as metabolic abnormalities, including insulin resistance, hypertension, and dyslipidemia (1,2). The diagnosis of this syndrome can be defined according to clinical, biochemical, and ultrasonography criteria (2).

Renal resistive index (RRI) is an ultrasound-based measurement that is potentially useful for the evaluation of acute and chronic kidney diseases (3,4). It is calculated as the following: peak systolic velocity/peak end diastolic velocity/peak systolic velocity (5). Additionally, RRI can provide prognostic data relating to systemic vasculature, and increased RRI is associated with hypertension, diabetes, and cardiovascular diseases (6,7).

The aim of this study was to investigate the RRI levels of patients with PCOS.

## SUBJECTS AND METHODS

This cross-sectional cohort study was carried out in the internal medicine outpatient clinics of a tertiary hospital in Turkey. The study was performed according to the terms of the ethical standards of the committee responsible for human experimentation and in accordance with the Declaration of Helsinki. Furthermore, the Institutional Review Board approved the study (04.11.2016/27). Informed consent was obtained from all subjects prior to their enrollment in this study.

A total of 257 female subjects, with a minimum age of 18 years, were enrolled in this study and divided into two groups. Thirty-four subjects were excluded due to a lack of radiological or biochemical tests, and seven subjects dropped out without stating a reason. As a result, the study group was composed of 109 patients with PCOS, which was diagnosed in accordance with the Rotterdam diagnostic criteria (8), and the control group consisted of 107 healthy subjects. The subjects' medical histories and conditions were carefully ascertained. Subjects with a history or prior diagnosis of chronic disease, metabolic disease, malignancy, clinical or biochemical features of Cushing syndrome, hyperprolactinemia, ovarian or adrenal virilizing tumors, or nonclassical congenital adrenal hyperplasia were excluded from this study. Breastfeeding, pregnant, and peri- or post-menopausal women were also excluded from this study.

Data collection for the purposes of this study included the following tests: demographic data, blood chemistry, blood pressure, and ultrasonography.

All women were dressed in lightweight clothes during the measurement of their weight. The women's height was measured in centimeters, and their weight was measured in kilograms. Body mass index (BMI) was calculated as the ratio of weight/height^2^ (kg/m^2^).

The patients' blood pressure was measured by trained nurses using periodically calibrated sphygmomanometers (Erka, Germany). Two separate measurements were performed for each subject.

A venous blood sample was collected in the morning following an overnight fast on the third day of the follicular phase of each subject's menstrual cycle. The women's fasting glucose, triglyceride, high-density lipoprotein (HDL), and low-density lipoprotein (LDL) levels were analyzed using a Roche C-501 (Tokyo, Japan), the hexokinase method (glucose), and a homogeneous colorimetric enzyme test (triglyceride, HDL, and LDL). Serum creatinine levels were analyzed on the Beckman Coulter Synchron LX-20 (Massachusetts, USA), using commercially available kits. Insulin levels were measured using the Abbott ARCHITECT i2000 SR analyzer system (Illinois, USA). Insulin resistance (IR) was measured using homeostasis model assessment [HOMA-IR = fasting glucose (mg/dL) x fasting insulin (μIU/mL)/405]. FSH and LH were measured using the ADVIA Centaur immunoassay system. Free testosterone levels were measured by radioimmunoassay (SRL Inc., Tokyo), and the reference range was between 1.1-4.7 pg/mL.

PCOS was diagnosed in cases in which two or more of the following three criteria were present: oligo- or anovulation, hyperandrogenism, and polycystic ovaries. Oligomenorrhea was defined as fewer than 10 menstrual cycles per year. Biochemical hyperandrogenism was determined on the basis of the patient's serum concentration of free testosterone. The criteria for the diagnosis of polycystic ovaries required the visualization of ≥ 12 follicles per ovary, which were 2 to 9 mm in diameter, or an ovarian volume > 10 cm^3^, according to transvaginal ultrasonography (8).

A transvaginal ultrasound examination was performed between days six and eight of the patient's menstrual cycle in order to assess ovarian morphology for the diagnosis of PCOS. Ultrasound examinations were performed using a 7-MHz transducer (General Electric Logic 400, Milwaukee, WI, USA).

RRIs of both kidneys were measured while the subject was in a supine position. All patients were examined in the morning following an overnight fast. An experienced radiologist measured the patients' RRI levels using GE's Logic 9 high-resolution color doppler ultrasonography (GE medical systems, Milwaukee, USA) with a 5-MHz transducer. Doppler signals were obtained from the interlobar arteries. The patients' RRI levels were calculated as peak systolic velocity/peak end diastolic velocity/peak systolic velocity.

Statistical analyses were performed using MedCalc Statistical Software version 16.8 (MedCalc, Belgium). The variables were investigated using both visual (histograms and probability plots) and analytical methods (the Kolmogorov-Smirnov test) to determine whether or not they were distributed normally. Categorical measurements were reported as numbers and percentages. Quantitative measurements were reported as the mean ± the standard deviation. The chi-square test was used to compare categorical measurements. The t-test or Mann-Whitney U tests were used in the comparison of quantitative measurements between the two groups. The correlation coefficient was used to analyze the degree of association between two variables [Pearson correlation coefficient (r) with p-value and 95% CI for r.]. A log transformation was used for variables that were not normally distributed. Multiple linear regression test (backward method) was used to analyse the relationship between a dependent variable (RRI) and one or more independent variables (age, systolic blood pressure, diastolic blood pressure, fasting glucose, insulin and HOMA). The probability of making a type I error (alpha, significance) is 0.05 in all tests.

## RESULTS

The groups were comparable according to age. The mean age of the patients with PCOS was 29.7 ± 3.4 while it was 29.6 ± 3.5 years in the healthy group (p = 0.790). Compared to the controls, the women with PCOS had a higher mean BMI (32.5 ± 7.0 vs. 29.3 ± 4.3 p < 0.001). The groups' demographic and laboratory data are shown in Table 1.

**Table 1:** Clinical and demographical data of the groups

	PCOS (N = 109)	Healthy (107)	p
Age	29.7 ± 3.4	29.6 ± 3.5	0.790
BMI (kg/m^2^)	32.5 ± 7.0	29.3 ± 4.3	< 0.001
Glucose (mg/dL)	91.7 ± 12.8	80.5 ± 10.5	< 0.001
HOMA-IR	4.8 ±4.4	1.6 ± 1.2	< 0.001
Insulin resistance N (%)	66 (60.5%)	9 (8.4%)	< 0.001
Systolic blood pressure (mmHg)	114.2 ± 10.8	110.8 ± 11.0	0.026
Diastolic blood pressure (mmHg)	71.1 ± 9.3	68.3 ± 10.7	0.039
Triglyceride (mg/dL)	117.1 ± 58.6	74.4 ± 23.9	< 0.001
LDL (mg/dL)	105.5 ± 27.2	68.2 ± 26.3	< 0.001
Creatinine	0.60 ± 0.13	0.58 ± 0.16	0.303

PCOS: polycystic ovary syndrome; BMI: body mass index; HOMA-IR: homeostasis model assessment of insulin resistance; LDL: low-density lipoprotein.

There were statistically significant differences in fasting glucose, insulin, HOMA-IR, triglyceride and LDL levels between the groups, with higher levels in patients with PCOS (p < 0.001, for each, respectively, Table 1). Moreover, systolic (114.2 ± 10.8 vs. 110.8 ± 11.0), and diastolic (71.1 ± 9.3 vs. 68.3 ± 10.7) blood pressures were high in patients with PCOS. The differences were statistically significant (p = 0.026, p = 0.039, respectively). Creatinine levels were comparable in both groups (p = 0.303).

Women with PCOS had significantly lower mean levels of FSH (5.0 ± 1.9 vs. 6.7 ± 2.6, p < 0.001). Conversely, they had significantly higher mean levels of LH (12.3 ± 8.6 vs. 6.8 ± 2.1, p < 0.001). Therefore, the LH/FSH levels of patients with PCOS were higher than those of the healthy subjects (2.4 ± 1.1 vs. 1.1 ± 0.5, p < 0.001). Compared to women in the control group, the women with PCOS had higher mean serum concentrations of free testosterone (5.4 ± 2.1 vs. 4.0 ± 1.7, p < 0.001). The frequency of polycystic over was found to be 88.1% (n = 96) in the study group, while it was 9.3% (n = 10) in the control group (Table 2).

**Table 2:** Comparison of the groups according to the hormonal and radiological data

	PCOS (N = 109)	Healthy (107)	p
FSH (mU/mL)	5.0 ± 1.9	6.7 ± 2.6	< 0.001
LH (mU/mL)	12.3 ± 8.6	6.8 ± 2.1	< 0.001
LH/FSH	2.4 ± 1.1	1.1 ± 0.5	< 0.001
Free testosterone (pg/mL)	5.4 ± 2.1	4.0 ± 1.7	< 0.001
Frequency of polycystic over N (%)	96 (88.1%)	10 (9.3%)	< 0.001
RRI	0.64 ± 0.06	0.57 ± 0.06	< 0.001

FSH: follicle stimulating hormone; LH: luteinizing hormone; RRI: renal resistive index.

The mean RRI was 0.64 ± 0.06 in patients with PCOS, and 0.57 ± 0.06 in healthy subjects. The patients with PCOS had significantly higher RRI levels than those in the healthy group (p < 0.001, Table 2).

**Figure 1:**
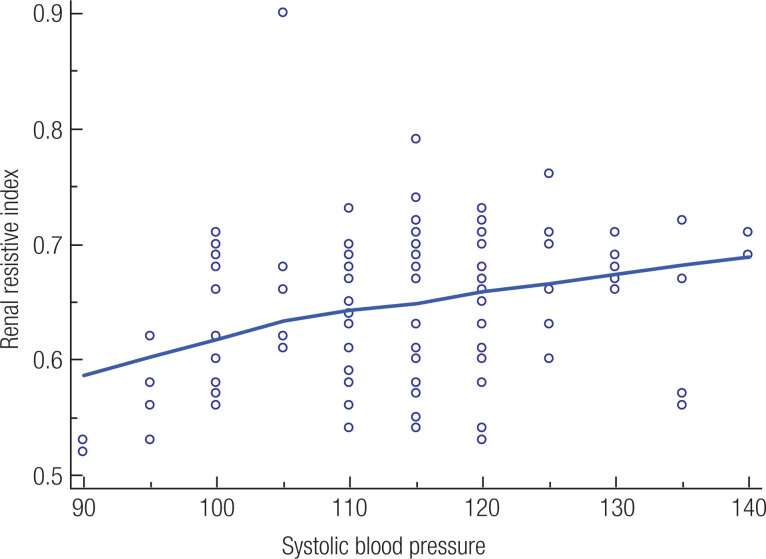
In a scatter diagram, the relation between renal resistive index and systolic blood pressure is presented graphically. One variable (the variable systolic blood pressure) defines the horizontal axis and the other (variable renal resistive index) defines the vertical axis. The values of the two variables on the same row in the data spreadsheet, give the points in the diagram.

**Figure 2:**
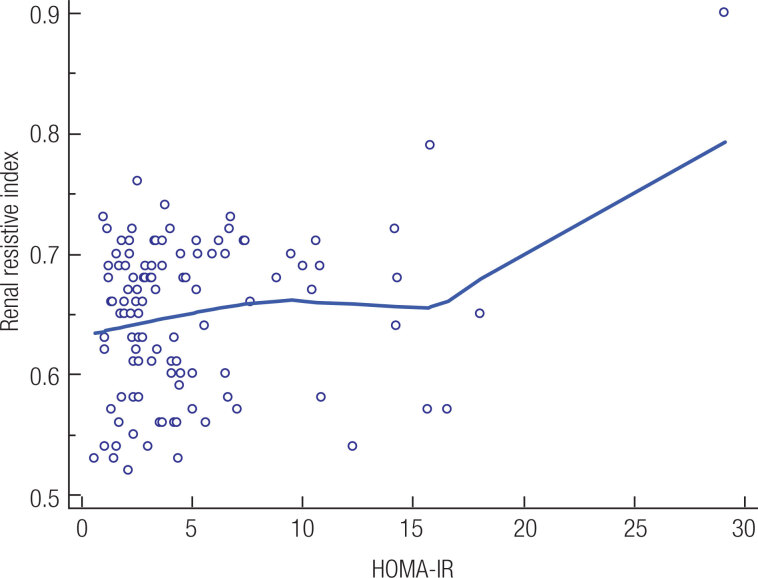
In a scatter diagram, the relation between renal resistive index and HOMA-IR is presented graphically. One variable (the variable HOMA-IR) defines the horizontal axis and the other (variable renal resistive index) defines the vertical axis. The values of the two variables on the same row in the data spreadsheet, give the points in the diagram. HOMA-IR: homeostasis model assessment of insulin resistance.

The RRI levels of patients with PCOS were correlated with systolic blood pressure (p = 0.0037, r = 0.276, Figure 1) and with HOMA-IR (p = 0.02, r = 0.216, Figure 2). Multiple regression analysis (backward method) was performed with RRI as a dependent variable and with systolic blood pressure, diastolic blood pressure, glucose, free testosteron and HOMA-IR as independent variables in both groups. A significant correlation persisted between RRI and systolic blood pressure (p < 0.004), and between RRI and HOMA-IR (p = 0.001) in patients with PCOS. On the other hand, no significant correlation persisted between RRI and systolic blood pressure (p = 0.489), and between RRI and HOMA-IR (p = 0.113) in healthy subjects.

## DISCUSSION

In this study, we investigated the RRI in patients with PCOS. We found higher levels of RRI in these patients when compared to healthy subjects. Moreover, we found a correlation between RRI and systolic blood pressure, and HOMA-IR in patients with PCOS. The resistive index measured in the intrarenal segmental arteries is a well-known marker of renal vascular and interstitial damage, corresponding to an increased total cardiovascular risk (9). Tedesco and cols. studied 566 patients with hypertension and reported that patients with higher RRI showed an increased left ventricular mass index and carotid intima-media thickness with a higher prevalence of left ventricular hypertrophy, carotid plaques and microalbuminuria. They concluded that RRI could predict the presence of early CV damage (10). Additionally, Keşkek and cols. reported that, as an independent risk factor for cardiovascular diseases, high level of homocysteine is also associated with high level of RRI in patients with hypertension (11). Furthermore, in the study by Liu and cols., 387 patients with DM were analyzed. They reported that higher level of RRI was found to be associated with microvascular complications in patients with diabetes (12). On the other hand, PCOS is one of the most common endocrinopathies in women with considerable negative effects on cardiovascular functions (13). These patients are generally insulin resistant, overweight and have metabolic syndrome, with arterial hypertension, dyslipidemia, impaired glucose tolerance and diabetes. Due to all these cardiovascular risk factors, patients with PCOS are in the high risk group for development of cardiovascular diseases. Moreover, women with PCOS and insulin resistance, high systolic blood pressure, overt vascular disease or kidney disease are in the very high-risk group (14,15). Therefore, high levels of RRI may be an indicator of cardiovascular and/or cardiovascular associated diseases in patients with PCOS.

In the current study, we found higher levels of fasting glucose, insulin and HOMA-IR in patients with PCOS. HOMA-IR is the most widely used as a surrogate measure of IR in large population studies and in the PCOS. Insulin resistance in PCOS contributes to both reproductive and metabolic disturbances (16). Therefore, it is clinically important to identify the prevalence and degree of IR in PCOS population. A defect in insulin receptor binding, phosphorylation or post-receptor insulin signaling transduction between the receptor kinase and glucose transport may lead to a decrease in insulin sensitivity in patients with PCOS and cause high blood glucose, insulin and HOMA-IR (17,18). Alebié and cols. compared 250 PCOS patients with 500 controls. They reported higher levels of HOMA-IR in patients with PCOS (19). In another study, Mu and cols.. found high levels of insulin resistance with adipose insulin resistance index in patients with PCOS (20).

High blood glucose, insulin, and HOMA-IR are also associated with high levels of RRI. Bruno and cols. compared 32 newly diagnosed type 2 diabetes patients with 27 healthy subjects. They reported higher RRI levels in patients with type 2 diabetes (21). Additionally, Trovato and cols. investigated 221 patients and concluded that, independent of nutrition, increased RRI is associated with higher degrees of insulin resistance (22).

In the present study, we found higher levels of systolic and diastolic blood pressures, triglyceride, LDL and BMI in patients with PCOS. Risk factors for cardiovascular system such as hypertension, diabetes, dyslipidemia and obesity are frequently reported in patients with PCOS (23). The main causes of high blood pressure in patients with PCOS may be insulin resistance, hyperandrogenism, greater sympathetic nerve activity and increased body weight (24). Shi and cols. compared 3396 PCOS patients with 1891 controls. They found higher prevalence of hypertension in patients with PCOS (19.2% vs. 11.9%) (25). Excess androgen levels, insulin resistance, variable amounts of estrogen exposure, and many environmental factors can cause to high triglyceride and LDL levels in patients with PCOS (26). Hong and cols. studied 507 Chinese patients with PCOS. They demonstrated significantly higher concentrations of triglyceride and LDL in patients with PCOS and insulin resistance (27).

Serum LH concentrations and the LH to FSH ratio are frequently elevated in women with PCOS (28). FSH levels are normal to slightly suppressed and do not increase to threshold levels required during the early follicular phase of the menstrual cycle to stimulate normal follicular maturation (29). In accordance with this link, when compared with the controls, serum FSH levels were low and LH levels were high in patients with PCOS in the present study.

This study has some limitations. First, it would have been beneficial if the clinical hyperandrogenism findings had been shown in addition to the biochemical hyperandrogenism. Second, for better understand the role of the RRI in PCOS patients, it would have been enlightening evaluate two more subgroups: women with hypertension without PCOS and women with insulin resistance without PCOS. This could not be done due to the wide exclusion criteria of the study. Further, the cross-sectional design of this study may be another limitation.

In conclusion, in the present study we found higher levels of RRI in patients with PCOS. High levels of RRI may be caused by cardiovascular risk factors such as high systolic blood pressure and insulin resistance. Furthermore, RRI can be a non invasive test for increased cardiovascular disease risks in patients with PCOS.
